# *Anopheles ziemanni* a locally important malaria vector in Ndop health district, north west region of Cameroon

**DOI:** 10.1186/1756-3305-7-262

**Published:** 2014-06-05

**Authors:** Raymond N Tabue, Thomas Nem, Jean Atangana, Jude D Bigoga, Salomon Patchoke, Frédéric Tchouine, Barrière Y Fodjo, Rose GF Leke, Etienne Fondjo

**Affiliations:** 1 National Reference Unit for Vector Control, The Biotechnology Center, University of Yaoundé I, P.O. Box 3851-Messa, Yaoundé, Cameroon; 2 Faculty of Science, Department of Biochemistry, University of Yaoundé I, P.O. Box 812, Yaoundé, Cameroon; 3 Ministry of Public Health, National Malaria Control Programme, P.O. Box 14386, Yaoundé, Cameroon

**Keywords:** Malaria, *Anopheles ziemanni*, *Anopheles gambiae*, Cameroon

## Abstract

**Background:**

Malaria transmission in Cameroon is mediated by a plethora of vectors that are heterogeneously distributed across the country depending on the biotope. To effectively guide malaria control operations, regular update on the role of local *Anopheles* species is essential. Therefore, an entomological survey was conducted between August 2010 and May 2011 to evaluate the role of the local anopheline population in malaria transmission in three villages of the Ndop health district in the northwest region of Cameroon where malaria is holoendemic, as a means to acquiring evidence based data for improved vector intervention.

**Methods:**

Mosquitoes were sampled both indoor and outdoor for four consecutive nights in each locality during each month of survey. Sampling was done by the human landing catch method on volunteers. *Anopheles* species were identified morphologically and their ovaries randomly dissected for parity determination. Infection with *Plasmodium falciparum* was detected by Circumsporozoite protein ELISA. Members of *An. gambiae* complex were further identified to molecular level by PCR and RFLP PCR.

**Results:**

*An. ziemanni* was the main malaria vector and whether outdoor or indoor. The man biting rate for the vectors ranged from 6.75 to 8.29 bites per person per night (b/p/n). The entomological inoculation rate for this vector species was 0.0278 infectious bites per person per night (ib/p/n) in Mbapishi, 0.034 ib/p/n in Mbafuh, and 0.063 ib/p/n in Backyit. These were by far greater than that for *An. gambiae*. No difference was observed in the parity rate of these two vectors. PCR analysis revealed the presence of only *An. colluzzi* (M- form).

**Conclusions:**

*An. ziemanni* is an important local malaria vector in Ndop health district. The findings provide useful baseline information on the anopheles species composition, their distribution and role in malaria transmission that would guide the implementation of integrated vector management strategies in the locality.

## Background

Malaria remains a serious health problem in sub-Saharan Africa, affecting mainly children less than five years old and expectant mothers [1]. In Cameroon about 40% of all deaths in children less than five years old are due to malaria [2]. Thanks to the intensifying efforts by control programmes in scaling-up the intervention strategies, the current global trends indicate a decline in the malaria morbidity and mortality by about fifty percent between 2000 and 2010 [1]. Nevertheless, this decrease in the number of malaria cases and deaths is not homogenous as most parts of the country remain highly endemic to malaria. This is linked to several factors which encompass the evolution and spread of drug resistance in the parasite, insecticide resistance in the vectors and the presence of a vast plethora of vectors species. These vectors are also unevenly distributed with differential efficiencies in mediating malaria transmission across the different endemic micro environments [3-9].

Knowledge of the vector profile in a given epidemiologic scenario is an important step to the planning and implementation of effective vector intervention strategies. Therefore, the acquisition of information on the local *Anopheles* population, their spatial distribution and contribution to malaria transmission is mandatory such that measures taken would be readily amenable to intervention.

About forty eight species of *Anopheles* have been identified in Cameroon [10-12] of which at least fourteen are reported to support the development and propagation of human *Plasmodia*. Amongst these are five major vectors, namely *Anopheles (cellia) gambiae* Giles, 1902; *Anopheles funestus* Giles, 1900; *Anopheles arabiensis* Patton, 1905; *Anopheles nili (Theobald), 1904; Anopheles moucheti moucheti* Evans, 1925 and at least nine secondary vectors (*Anopheles paludis* Theobald, 1900*; Anopheles carnavalei* Brunhes, le Goff & Geoffroy, 1998; *Anopheles coustani Laveran,* 1900*; Anopheles marshallii (Theobald), 1903; Anopheles ziemanni Gruenberg, 1902; Anopheles pharoensis* Theobald, 1901; *Anopheles hancocki,* Edwards 1929; *Anopheles wellcomei wellcomei* Theobald, 1904; *Anopheles (cellia) ovengensis*) [13-17]. To date, there is insufficient knowledge on the bionomic and role of these secondary vectors in the malaria transmission in many areas of the country. Their occurrence, abundance and composition tend to vary greatly with the eco-epidemiological setting. In fact, Cameroon is characterized by a southern forested equatorial zone where transmission is perennial, a northern Sudanian savannah zone with a long (five to six months) seasonal transmission pattern, and a sahelian savannah zone of the far north with very short (about 3 months) seasonal transmission [2,3,5]. Depending on the setting, the vectors may be sympatric or occur in isolation mediating transmission either at the same time or at different times.

Ndop health district is a specific ecological area in the north western savannah of Cameroon. It is characterized by paddy fields that are irrigated by water sourcing from numerous natural lakes in the vicinity. Such lakes are known to be prolific for *Anopheles* (especially *An. ziemanni*) breeding that upholds transmission in such locality. However, there is no existing information on the mosquito species composition and their role in malaria transmission in Ndop. Therefore, this study sought to primarily characterize the mosquito fauna and determine the role of anophelines in the transmission of malaria in the locality.

## Methods

### Description of the study site

**Figure 1:**
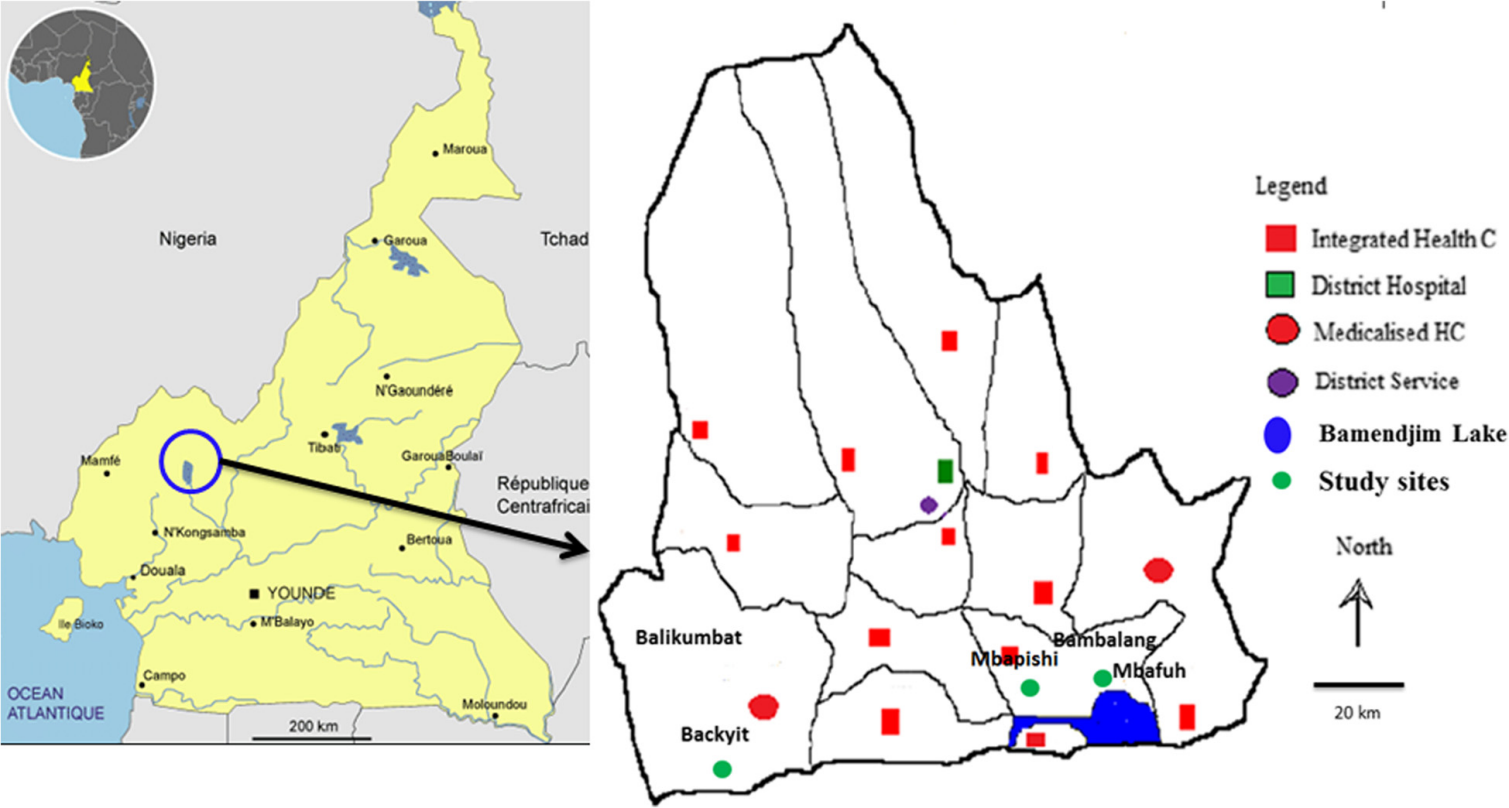
Map of Ndop Health District showing the location of the study sites.

The study was carried out in Ndop Health District, situated 06°00'N, 10°42'E in the Northwest Region of Cameroon (Figure 1). The climate is characterized by a long rainy season (June to November) with rainfall averaging 1800–2500 mm per year, average relative humidity of 97% to 98% and mean annual temperature of 24°C. Cross sectional surveys were carried out during the peak of the rainy season (August-November) and during three months of the dry season (January, April and May). Three villages in Ndop Health District were selected: Mbafuh, Mbapishi and Backyit.

Mbafuh and Mbapishi belong to Bambalang health area and are characterized by their proximity to the Bamendjim Lake. This lake creates wetlands that are actively occupied for agriculture. Its population is estimated at 19,000 inhabitants dispersed in 4 villages. Backyit is the main village of Balikumbat health with a population of about 23,133 inhabitants. In these three villages, agriculture is the main economic activity with rice cultivation being commonest practice in the wetlands of its plains.

### Adult mosquito sampling

Mosquito sampling took place simultaneously in the three villages belonging to two health areas (Backyit in Balikumbat health area, Mbafuh and Mbapishi in Bambalang health area). Human landing collections were performed during four consecutive nights from 06:00 pm-06:00 am each month. Mosquitoes were collected indoors and outdoors in three randomly selected houses (at least 50 m apart) and rotating between houses at different locations in each village each night. A team of two trained volunteers per house; one sitting inside the house and the other on the veranda collected female mosquitoes as they landed on exposed lower limbs, making a total of 24 human-nights per month per village. The volunteers changed collection points (indoor or outdoor) every two hours in order to minimize bias. Ethical clearance was obtained from the National ethics committee of Cameroon (N°: FWA IRB00001954). Consent from household heads was sought prior to using the house for mosquito collection. Participation in mosquito collection was strictly voluntary and only those adequately trained on the collection procedures were retained.

### Field processing of mosquitoes

*Anopheles* mosquitoes collected were sorted and identified morphologically using the identification keys of Gillies and de Meillon [18] and Gillies and Coetzee [19]. Anopheline species were randomly dissected and the ovaries examined to determine parity as described previously [20]. The carcasses of both the dissected and non-dissected anopheline mosquitoes were individually stored desiccated in labeled Eppendorf tubes containing silica gel for subsequent laboratory analyses.

### Laboratory processing of anophelines

The head-thorax portion of each mosquito was homogenized in blocking buffer (0.5% Casein, 0.1 N NaOH, 1 × PBS) and used to check for the presence of *Plasmodium falciparum* Circumsporozoite antigen (CSA) by ELISA previously [21,22]. A positive control (Kikergaard & Perry Laboratories, USA) and negative controls (uninfected laboratory reared mosquitoes) were tested along with the rest of the samples. A sample was considered positive if the optical density of the resulting green coloration visualized at 405 nm was greater than the mean of those of the negative controls plus three times the standard deviation. The head-thorax portions of the mosquitoes were used to guard against overestimating the infection rates.

Mosquitoes belonging to *An. gambiae s.l* were randomly selected for molecular identification. DNA was extracted from the legs and wings of selected mosquitoes by the method described by Collins *et al*., 1987 [23]. The extracted DNA was suspended in 25 μl of sterile TE buffer (10 mMTris - HCl pH 8.1, 1 mM EDTA) and used to identify *An. gambiae* siblings by PCR [24]. The presence of the M and S molecular forms of *An. gambiae s.s* was determined by restriction fragment length polymorphism (RFLP) of ribosomal DNA [22].

### Data analysis

Data of the four consecutive nights of sampling in each village and per month were calculated. The following entomological indices were calculated: the man biting rate (m.a), which represents the average number of bites received per person per night; the Infection Rate (IR), that measured the proportion of mosquitoes positive for *P. falciparum* CSA by ELISA; the Parity rate, which is the ratio of parous mosquitoes to the overall dissected; Entomological Inoculation Rate (EIR) which is the number of infective bites received per person per night; EIR is calculated as the product of the m.a and IR. Data were analyzed using SPSS Statistics software version 17.0 and the Kruskal Wallis test was used to compare means at 95% confidence interval.

## Results

### Mosquito composition

A total of 33,739 mosquitoes were collected during our study. The proportion of anophelines collected from the three villages ranged from 10.23% to 13.26% (Table 1). Nuisance was mainly caused by *Aedes Sp, Coquillettidia Sp, Culex Sp,* and *Mansonia Sp*. The density of *Mansonia Sp* was very high in the three villages and varied from 45.9% to 83.47% of the total culicines collected. *Mansonia Sp* and *Culex Sp* were the dominant culicine mosquitoes in the three study sites with a highest density of 83.47% observed in Mbafuh (Table 1). *Aedes Sp* was present at a low density and only 112 mosquitoes were collected, none was collected from Mbapishi. *Coquillettidia Sp* was also present, but at low densities. *Anopheles* species represented 12.05%, 10.23% and 13.26% of the total mosquito population in Backyit, Mbafuh, and Mbapishi respectively.

**Table 1:** Composition of the Culicine fauna in Backyit, Mbafuh and Mbapishi

	**August 2010**	**September 2010**	**October 2010**	**November 2010**	**January 2011**	**April 2011**	**May 2011**	**Total**	**Percentage**
**Backyit**									
*Anopheles Sp*	104	144	380	644	217	8	55	1552	12.05%
*Aedes Sp*	16	14	0	0	0	7	72	109	0.85%
*Coquillettidia Sp*	0	0	0	5	1	0	2	8	0.06%
*Culex Sp*	1763	877	841	408	410	236	765	5300	41.14%
*Mansonia Sp*	264	1101	1360	1357	338	370	1124	5914	45.91%
Total	2147	2136	2581	2414	966	621	2018	12883	100.00%
**Mbafuh**									
*Anopheles Sp*	11	150	366	379	241	9	12	1168	10.23%
*Aedes Sp*	0	1	1	0	0	0	0	2	0.02%
*Coquillettidia Sp*	2	6	16	11	4	1	0	40	0.35%
*Culex Sp*	204	117	93	50	195	8	10	677	5.93%
*Mansonia Sp*	992	1693	2022	1695	1265	416	1443	9526	83.47%
Total	1209	1967	2498	2135	1705	434	1465	11413	100.00%
**Mbapishi**									
*Anopheles Sp*	96	237	375	237	233	54	20	1252	13.26%
*Aedes Sp*	0	0	0	0	0	0	0	0	0.00%
*Coquillettidia Sp*	0	4	12	1	5	1	0	23	0.24%
*Culex Sp*	268	126	137	167	101	3	10	812	8.60%
*Mansonia Sp*	953	1235	1110	1571	1063	251	1173	7356	77.90%
Total	1317	1602	1634	1976	1402	309	1203	9443	100.00%

### Anopheles diversity and abundance in the study localities

The anopheline population collected varied from one site to another and some species were common to all the sites. In the three villages, *An. ziemanni* and *An. gambiae s.l* were the dominant vector species. A total of 187 *An. gambiae* as compared to 3697 *An. ziemanni* were collected in all three sites (Table 2).

**Table 2:** Anopheles diversity and abundance

	**August 2010**		**September 2010**		**October 2010**		**November 2010**		**January 2011**		**April 2011**		**May 2011**		**Total**		**Total**
**Anopheline species**	**Outdoor**	**Indoor**	**Outdoor**	**Indoor**	**Outdoor**	**Indoor**	**Outdoor**	**Indoor**	**Outdoor**	**Indoor**	**Outdoor**	**Indoor**	**Outdoor**	**Indoor**	**Outdoor**	**Indoor**	**Outdoor + Indoor**
**Backyit**																	
*An. gambiae s.l.*	21	35	19	14	1	6	2	2	3	0	4	3	7	5	57	65	122 (7.86%)
*An. ziemanni.*	24	20	55	54	174	199	367	273	111	79	1	0	25	12	757	637	1394 (89.82%)
*An. christyi*	2	0	0	0	0	0	0	0	0	0	0	0	0	0	2	0	2 (0.13%)
*An. implexus*	1	1	0	1	0	0	0	0	15	7	0	0	2	2	18	11	29 (1.87%)
*An. nili*	0	0	1	0	0	0	0	0	0	0	0	0	0	0	1	0	1 (0.06%)
*An. maculipalpis*	0	0	0	0	0	0	0	0	1	1	0	0	1	1	2	2	4 (0.26%)
**TOTAL**	48	56	75	69	175	205	369	275	130	87	5	3	35	20	837	715	1552
**Mbafuh**																	
*An. gambiae s.l.*	0	0	1	1	0	0	0	0	0	1	0	0	3	2	4	4	8 (0.68%)
*An. ziemanni.*	6	5	85	63	200	163	190	188	103	131	0	0	0	0	584	550	1134 (97.09%)
*An. christyi*	0	0	0	0	0	0	0	0	0	0	5	3	4	2	9	5	14 (1.20%)
*An. implexus*	0	0	0	0	0	0	1	0	3	2	0	0	0	0	4	2	6 (0.51%)
*An. tenebrosus*	0	0	0	0	1	2	0	0	0	1	0	1	0	0	1	4	5 (0.43%)
*An. funestus*	0	0	0	0	0	0	0	0	0	0	0	0	1	0	1	0	1 (0.09%)
**TOTAL**	6	5	86	64	201	165	191	188	106	135	5	4	8	4	603	565	1168
**Mbapishi**																	
*An. gambiae s.l.*	1	1	0	0	0	0	0	0	0	1	50	0	1	3	52	5	57 (4.55%)
*An. ziemanni.*	56	35	134	102	272	99	158	69	153	71	3	1	9	7	785	384	1169 (93.37%)
*An. christyi*	1	0	1	0	3	1	7	1	7	1	0	0	0	0	19	3	22 (1.76%)
*An. implexus*	0	0	0	0	0	0	1	1	0	0	0	0	0	0	1	1	2 (0.16%)
*An. pharoensis*	1	1	0	0	0	0	0	0	0	0	0	0	0	0	1	1	2 (0.16%)
**TOTAL**	59	37	135	102	275	100	166	71	160	73	53	1	10	10	858	394	1252

In Backyit, 06 *Anopheles* species were encountered. *An. ziemanni* represents 90% of anopheline species collected while *An. gambiae* represented 8%*.* The other species (*An. funestus, An. christyi, An. pharoensis, An. moucheti, An. rufipes,* and *An. implexus*) represented just 2% of this population. About 97% and 93% of anopheline species collected respectively in Mbafuh and Mbapishi was composed of *An. ziemanni* (Table 2). In total, 12 *Anopheles* species were collected in the three villages with a high density of *An. ziemanni* that varied from 89.82% to 97.09%. This was followed by *An. gambiae s.l*. Other species were either absent or were at low densities, varying from one village to another. PCR analysis of *An. gambiae* complex revealed that the entire *An. gambiae s.l* population was made up of *An. gambiae s.s*. and only the M form was present in the three villages.

### Variation in the biting rate, feeding behavior and entomological inoculation rate of *An. gambiae and An. ziemanni*

**Figure 2:**
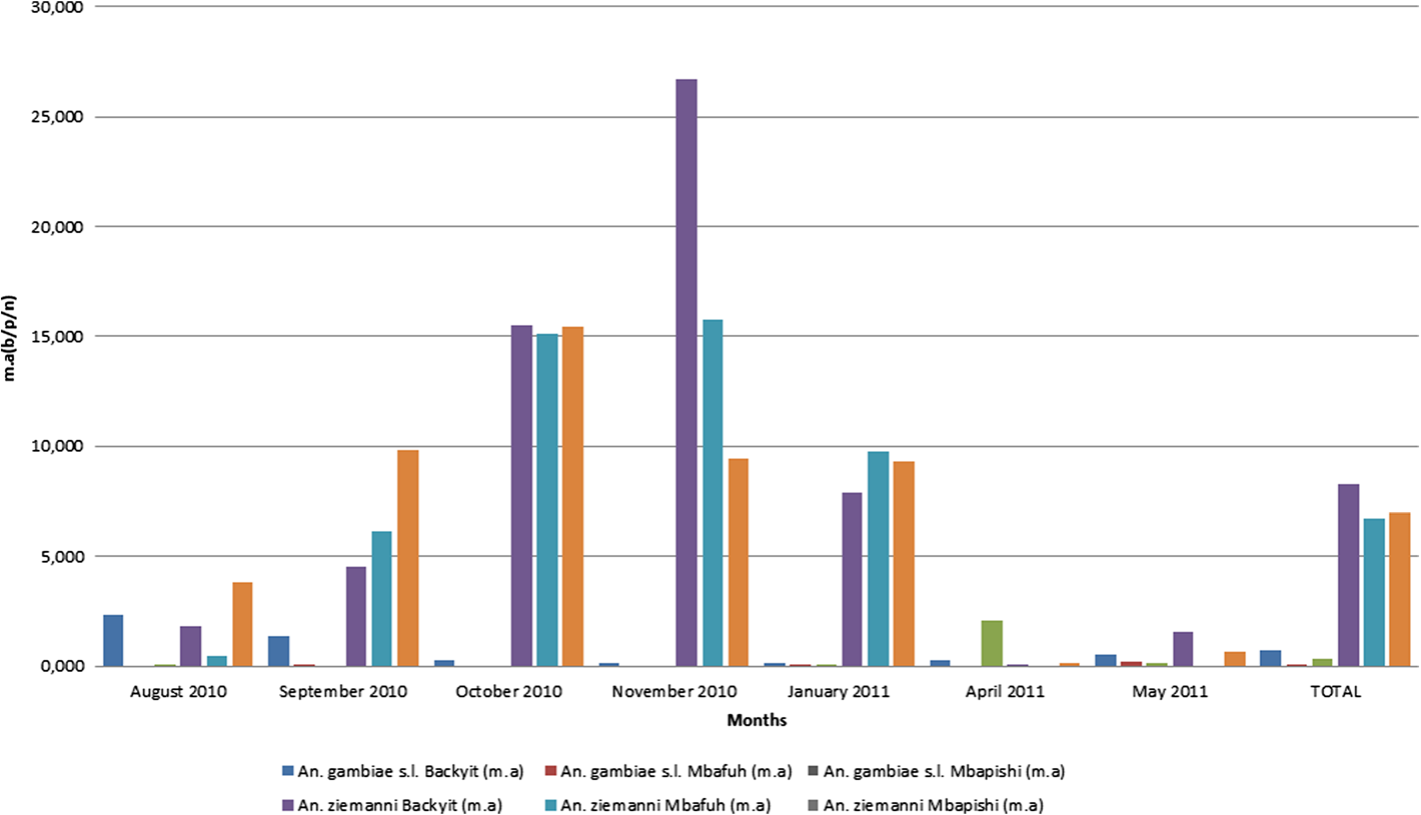
**Anopheline man biting rate in Backyit, Mbafuh and Mbapishi.** m.a: man biting rate, b/p/n: bites per person per night.

*Anopheles ziemanni* was the most aggressive species with a man-bite rate of 8.29 bites per person per night (b/p/n) in Backyit, 6.75 b/p/n in Mbafuh and 6.96 b/p/n in Mbapishi (Figure 2). When compared to *An. gambiae*, the difference in aggressivity in the three villages was significant (p < 0.05). However, the man biting rate of *An. gambiae* was at times higher than that of *An. ziemanni* (August 2010, January 2011 in Backyit and April 2011 in Mbapishi) (Table 3).

**Table 3:** **Variation in the Entomological Inoculation Rate (EIR) for *An. gambiae and An. ziemanni***

	**August 2010**				**September 2010**				**October 2010**				**November 2010**				**January 2011**				**April 2011**				**May 2011**				**Total**			
	**N**	**m.a**	**IR**	**EIR**	**N**	**m.a**	**IR**	**EIR**	**N**	**m.a**	**IR**	**EIR**	**N**	**m.a**	**IR**	**EIR**	**N**	**m.a**	**IR**	**EIR**	**N**	**m.a**	**IR**	**EIR**	**N**	**m.a**	**IR**	**EIR**	**N**	**m.a**	**IR**	**EIR**
**Backyit**																																
*An. gambiae s.l.*	56	2.333	0.02	0.047	33	1.375	0	0	7	0.292	0	0	4	0.167	-	-	3	0.125	0	0	7	0.292	0	0	12	0.5	0	0	122	0.726	0.011	0.008
*An. ziemanni*	44	1.833	0.026	0.048	109	4.542	0	0	373	15.542	0.005	0.078	640	26.667	0.006	0.16	190	7.917	0	0	1	0.042	0.08	0.003	37	1.542	0.04	0.062	1394	8.298	0.007	0.058
**Total**	100	4.167	0.022	0.092	142	5.917	0	0	380	15.833	0.005	0.079	644	26.833	0.006	0.161	193	8.042	0	0	8	0.333	0.03	0.01	49	2.042	0.03	0.061	1516	9.024	0.007	0.063
**Mbafuh**																																
*An. gambiae s.l.*	0	0	0	0	2	0.083	0	0	0	0	-	-	0	0	-	-	1	0.042	0	0	0	0	-	-	5	0.208	0	0	8	0.333	0	0
*An. ziemanni*	11	0.458	0	0	148	6.167	0	0	363	15.125	0.008	0.121	378	15.75	0.005	0.079	234	9.75	0.004	0.039	0	0	-	-	0	0	0	0	1134	6.750	0.005	0.034
**Total**	11	0.458	0	0	150	6.25	0	0	363	15.125	0.008	0.121	378	15.75	0.005	0.079	235	9.792	0.004	0.039	0	0	-	-	5	0.208	0	0	1142	6.798	0.005	0.034
**Mbapishi**																																
*An. gambiae s.l.*	2	0.083	0	0	0	0	-	-	0	0	-	-	0	0	-	-	1	0.042	-	-	50	2.083	-	-	4	0.167	0	0	57	0.339	0	0
*An. ziemanni*	91	3.792	0	0	236	9.833	0	0	371	15.458	0.008	0.124	227	9.458	0.004	0.038	224	9.333	0.004	0.037	4	0.167	0	0	16	0.667	0	0	1169	6.958	0.004	0.028
**Total**	93	3.875	0	0	236	9.833	0	0	371	15.458	0.008	0.124	227	9.458	0.004	0.038	225	9.375	0.004	0.038	54	2.25	0	0	20	0.833	0	0	1226	7.298	0.004	0.029

**N**: number of mosquitoes collected; **m.a**: man biting rate (aggressivity); **IR**: Infection Rate; **EIR**: Entomological Inoculation Rate.

**Figure 3:**
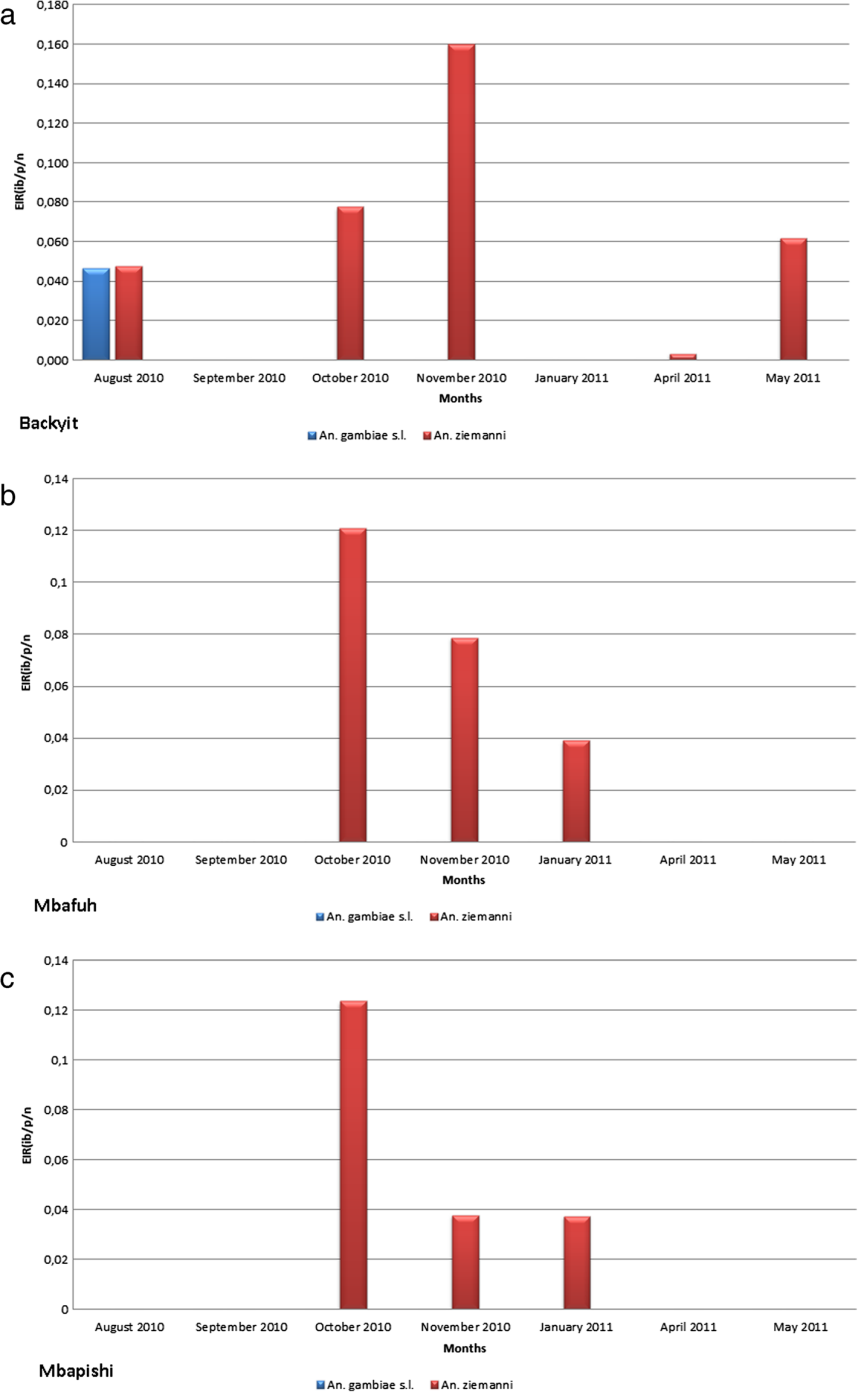
**An. ziemanni and An. gambiae EIR variation in Backyit, Mbafuh and Mbapishi. a**. EIR variation of An. ziemanni and An. gambiae in Backyit. **b** - EIR variation of An. ziemanni and An. gambiae in Mbafuh. **c** - EIR variation of An. ziemanni and An. gambiae in Mbapishi.

Table 3 shows the variation in the entomological inoculation rates (EIR) of the two local vectors species during the survey months. The number of infectious bites a person would receive per night was observed to vary between 0.028 ib/p/n and 0.058 ib/p/n for *An. ziemanni*. While the EIR for *An. gambiae s.l* was 0.008 ib/p/n in Backyit (Figure 3a), this species did not contribute to transmission in the other villages. Backyit also recorded the highest combined EIR for *An. gambiae* and *An. ziemanni* during the survey compare to Mbafuh and Mbapishi (Figure 3b, c).

**Figure 4:**
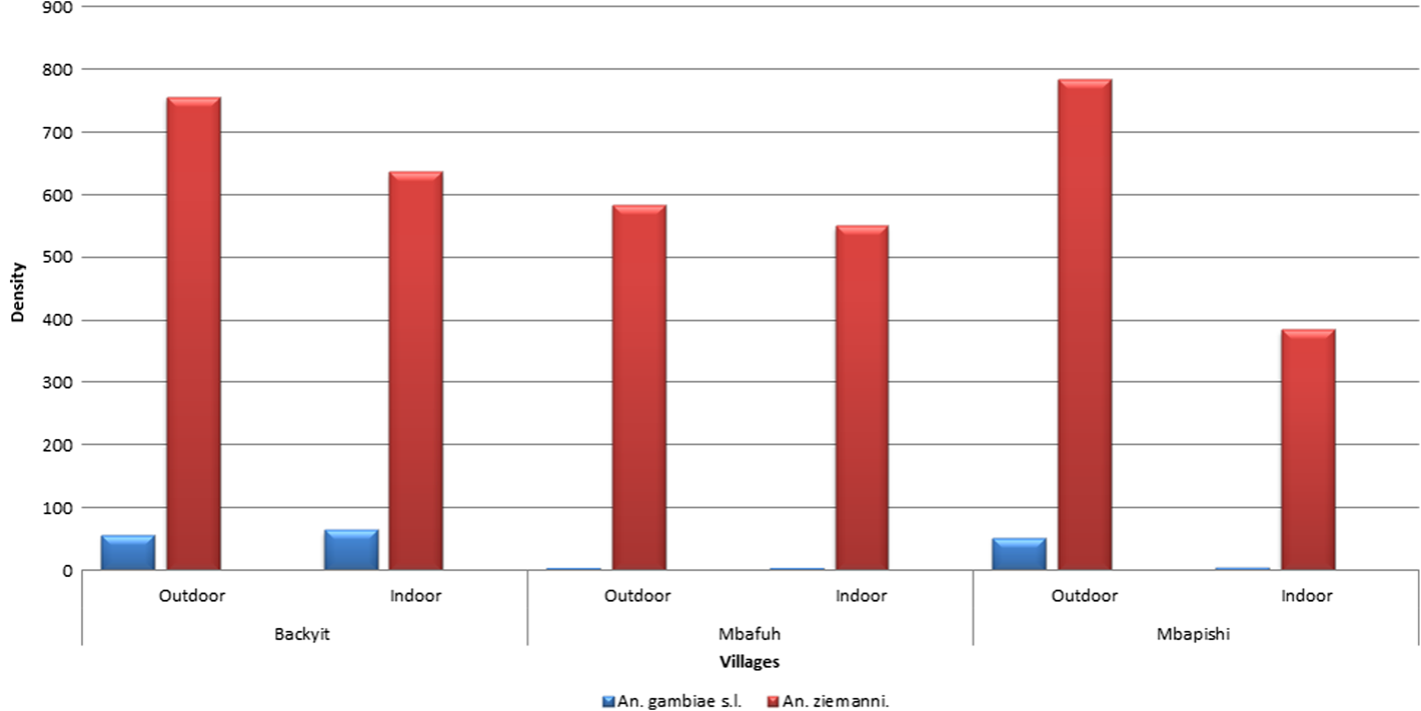
**Biting behavior of *An. ziemanni and An. gambiae *** **in Backyit, Mbafuh and Mbapishi.**

The biting behaviour of the two species varied between the villages and from one month to another. In general, there was no difference in the biting preference for *An. ziemanni* and *An. gambiae s.l* in Backyit and Mbafuh (Figure 4). The difference was however higher for the two species in Mbapishi where they were strongly exophagic. In general, *An. ziemanni* had the highest density in all three study sites and was observed to bite mainly outdoor compared to indoor. This difference was more pronounced in Mbapishi where the density was 785 outdoor and 389 indoor for *An. ziemanni* and 52 outdoor and 5 indoor for *An. gambiae s.l* (Figure 4). The difference in indoor and outdoor proportion of *An. ziemanni* in the three villages was significant (p < 0.05).

Following the dissection and examination of the ovaries, *An. gambiae* and *An. ziemanni* showed high parity rates in all three villages. However, the low density of *An. gambiae* did not allow for any statistical comparison of its parity to that of *An. ziemanni.* For the two vector species*,* the parity rate varied between 61.11% and 93.02% in Backyit, 39.93% and 78.05% in Mbafuh and from 19% and 81% in Mbapishi. The monthly variations were not significant (p > 0.05).

## Discussion

Successfully controlling malaria compels the implementation of effective vector intervention strategies. Because of climate change and vector dynamics, it is required that a continuous update on the vectors be made, to assess their contribution to malaria transmission such that measures taken are readily amenable for intervention in any given epidemiological setting. The present study aimed at acquiring baseline information on the spatial distribution and contribution of the malaria vectors to transmission in the health district of Ndop, in the Northwest region of Cameroon.

Studies in most parts of the equatorial forested areas of Cameroon, *An. gambiae s.l* have been reported as the main vector species with playing only minor secondary role in malaria transmission. For the first time *An. ziemanni* is portrayed here to be the primary vector species with *Anopheles gambiae* relegated to playing the role of a secondary vector in the Ndop health district. Generally, the densities of mosquitoes collected during the peak of rainy season are more important compare to the dry season. This can be example by the number of breeding site more important during the rainy season. The identification of up to twelve different Anopheles species in this locality is not surprising and only confirms the extensive and complex plethora of the *Anopheles* fauna already demonstrated and thriving in varied ecological settings of the country [5,11,25,26]. Thus, the results show that *An. ziemanni* was the most abundant *Anopheles* species during the period of the study in the different sites and is responsible for maintaining transmission even in the absence of the other vectors; this phenomenon is analogous to observations in western Kenya where in the presence of other vectors, *An. ziemanni* were found have the highest *Plasmodium falciparum* infection rates [27]. The high density of *An. ziemanni* was probably a consequence of the ecosystem that enables the proliferation of breeding sites contributing to its development. Indeed, a study to assess the density of breeding sites in these villages revealed that the surroundings natural lakes and other flowing streams were suitable breeding sources for *An. ziemanni*. It turned out that the selected sites were located near lakes created by the Bamendjim dam.

Overall, the transmission intensity was low as shown by the entomological inoculation rates, despite high density of *An. ziemanni*, the major vector species in the three villages. This is likely due to the high LLIN coverage (67.3%) in the region after the mass campaign and free distribution of LLIN [2]. Indeed, several studies across sites in Africa have demonstrated the efficacy of LLIN to reduce the malaria burden and transmission at the community level [28]. However, though not uncommon, this low transmission despite the high parity rates observed might be due to low vector anthropophily, implying *An. ziemanni* may be feeding on hosts other than human. This will be investigated further. This species however, has been previously reported to play a secondary role in the transmission of malaria in several eco-epidemiological settings of Cameroon [5,29]. *An. gambiae s.l* generally known to be the main malaria vector in most areas of the southern forested parts of the country [30,31] was found in low numbers played only minor secondary role in malaria transmission in Ndop. Although Ndop is situated in the mid-western highlands that paves into the northern savannah regions, no *An. arabiensis* was found. Only *An. gambiae s.s,* M molecular form was found in the three villages corroborating with as previously reported finding in other forested areas of African [4,32,33]. This species is also the most widespread and major malaria vector in Cameroon and elsewhere in Africa [8,25,34-39].

The study revealed very high parity rates within the anopheline populations, implying that the vectors survive long enough to support the extrinsic sporogonic cycle of *Plasmodium* and therefore are able to transmit and even re-transmit malaria after several cycles of feeding on humans. Considering the good LLIN coverage (about 67.3%), this might have led to a change in the vector behavior becoming more exophilic and thus escaping the LLIN. This in part might have contributed to the apparently high parity rates. The *Culicidae* nuisance is provided mainly by the *Aedes, Coquillettidia, Culex*, and *Mansonia* species. The breeding sites created by pigs in nearby households particularly favored the proliferation of the culicines.

## Conclusion

Environmental pressures and climate change bring about malaria vectors dynamism, which leads to some malaria vectors becoming more efficient in transmitting malaria [40]. As a perfect illustration of this vector dynamism, it seems that *An. ziemanni* could sustain the transmission of malaria on its own in Ndop health district even in the absence of *An. gambiae s.l*, a major vector species in most parts of the country. Pending further investigation, these results provide useful baseline information on the heterogeneity in anopheline species composition and distribution maps that should be taken into account in vector control operations in Cameroon.

## Competing interests

The authors declare that they have no competing interests.

## Authors' contributions

EF conceived and planned the study and its design. RNT, TN, SP, JA, FT monitored the field and laboratory studies, analyzed and interpreted the data. RT, JB, EF, RL drafted the manuscript and, BF involved in the coordination of the laboratory studies and was involved in field and laboratory work for acquisition. JB assisted in the molecular and infectivity analyses. JB, EF and RL critically read the manuscript. All authors read and approved the final manuscript.
